# Modified hypothermic circulatory arrest for emergent repair of acute aortic dissection type a: a single-center experience

**DOI:** 10.1186/1749-8090-8-125

**Published:** 2013-05-09

**Authors:** Hong Qian, Jia Hu, Lei Du, Ying Xue, Wei Meng, Er-yong Zhang

**Affiliations:** 1 Department of Cardiovascular Surgery, West China Hospital, Sichuan University, Chengdu, People’s Republic of China; 2 Department of Anesthesiology, West China Hospital, Sichuan University, Chengdu, Sichuan, People’s Republic of China

**Keywords:** Acute aortic dissection, Hypothermia, Circulatory arrest, Aortic surgery

## Abstract

**Background:**

Deep hypothermic circulatory arrest (DHCA) with antegrade cerebral perfusion has been historically preferred for organ protection during surgical repair of the acute aortic dissection type A. However, in the past decades, different perfusion-specific strategies with a growing trend to increase the body temperature at circulatory arrest emerged. In this study, we retrospectively analyzed the clinical results of our modified protocol for cardiopulmonary bypass and hypothermia management.

**Methods:**

Between February 2007 and September 2012, 54 consecutive patients suffering from acute aortic dissection type A underwent emergent surgery. All patients received hypothermic circulatory arrest in combination with antegrade cerebral perfusion. The patients were divided into two subsets according to the degree of hypothermia and perfusion strategies: namely the DHCA group and the group of modified hypothermic circulatory arrest (MHCA).

**Results:**

The overall 30-day mortality was 27.8% and was not significantly different between groups (DHCA, 33.3%, MHCA, 19%; *p*=0.253). The requirement for blood product transfusion in MHCA patients was significantly less as as compared with the patients in the DHCA group. No difference occurred in the incidence of temporary neurologic dysfunction, dialysis-dependent renal failure, or reexploration for bleeding between two groups of patients. The use of MHCA was identified as a protective factor against the postoperative composite complications (OR, 0.78; CI, 0.52 to 0.98; *p*=0.04) and the prolonged intensive care unit stay (OR, 0.8; 95% CI, 0.56 to 0.98; *p*=0.04).

**Conclusions:**

Moderate hypothermia in combination with selective brain perfusion and systemic retrograde perfusion is associated with adequate cerebral and visceral protection, reduced postoperative complications and shortened intensive care unit stay in our series. This modified perfusion strategy may help in improving perioperative outcomes in this particular group of patients.

## Background

Acute aortic dissection type A (AADA) is one of the most challenging diseases in cardiovascular surgery. Despite the continuous advances of operative techniques and perfusion-specific technologies during recent years, several international registries still reported an in-hospital mortality rate in patients with AADA of 17%-28% [1-4]. Ischemic visceral organ failure and postoperative neurologic dysfunction are closely associated with the significant perioperative mortality and morbidity in surgically treated patients. During past decades, different management of the circulation for the cerebral and visceral organ protection during complicated aortic reconstruction has been developed, however the optimal strategy remains the subject of ongoing debate [3,5].

Deep hypothermic circulatory arrest (DHCA) with selective antegrade cerebral perfusion (ACP) is an effective cerebral protection technique and has widely been applied in the surgical repair of AADA requiring open distal anastomosis. More recently, the absolute necessity for deep hypothermia during circulatory arrest once physiologic ACP is provided has been questioned. A series of studies have demonstrated that patients undergoing mild or moderate hypothermic circulatory arrest were presented with a reduction in the duration of cardiopulmonary bypass (CPB), systemic inflammatory responses, organ dysfunction and coagulation disorders [6-10]. However, concerns also grew among the surgeons about the effectiveness of the mild-to-moderate hypothermia for protecting visceral organs and lower body during circulatory arrest, especially when a long arch repair time was required [2,11,12].

We herein present the early results of different perfusion strategies that have been employed at our institution during emergent repair of AADA over a five-year period. Our cerebral and visceral organ protection protocol evolved from the use of DHCA (core temperature < 25°C) with unilateral ACP to modified hypothermic circulatory arrest (MHCA) over the study period. The MHCA protocol consists of moderate hypothermia (core temperature 25°C-28°C) and unilateral ACP combined with episodes of retrograde systemic perfusion during circulatory arrest. This change of paradigm allows us to retrospectively review and assess the safety and outcomes of performing AADA repair with these two perfusion-specific techniques.

## Methods

### Patient profile

Data from two hundred and twenty-two patients with AADA who admitted to our institution between February 2007 and September 2012 were prospectively collected and retrospectively analyzed. Of these patients, 201 patients (90.2%) were treated surgically, and only 54 patients underwent emergent repair with the aid of hypothermic circulatory arrest, selective ACP. Reasons for turning down from emergent surgery were advanced age, comorbidity and patient refusal. Retrospective data analysis was approved by the Institutional Review Board at West China Hospital in compliance with the Declaration of Helsinki. The need for individual patient consent was waived. The patients were divided into two subsets according to the perfusion protocol during circulatory arrest: namely the DHCA group and the MHCA group. Perioperative characteristics of the patients in both groups are summarized in Table 1.

**Table 1:** Preoperative demographics and clinical characteristics

**Risk factors**	**DHCA**	**MHCA**	***p *Value**
	**(n=33)**	**(n=21)**	
Age, years	44.9±11.7	46.9±10.7	0.533
Weight, kg	68.3±11.6	67.0±11.3	0.669
Male/Female	26/7(78.8%/21.2%)	17/4(81.0%/19.0%)	1.000
Smoking history	15 (45.5%)	10 (47.6%)	0.876
Hypertension	14 (42.4%)	8 (38.1%)	0.752
Diabetes	8 (24.2%)	5 (23.8%)	0.971
Etiology			
Aneurysmal disease	28 (84.8%)	19 (90.5%)	0.693
Marfan syndrome	4 (12.1%)	1 (4.8%)	0.638
Others	1 (3.0%)	1 (4.8%)	1.000
Mitral regurgitation	2 (6.1%)	0 (0%)	0.516
Aortic regurgitation	16 (48.5%)	8 (38.1%)	0.638
ASA classification	E3 (E2-E5)	E4 (E3-E5)	0.606
LVEF	64.4%± 5.3%	63.4% ± 8.9%	0.629
Hemoglobin (g/L)	124.9±17.0	123.9±19.4	0.847
Hematocrit (%)	0.38±0.05	0.37±0.05	0.733
White blood cell (10 [9]/L)	9.6±3.1	8.2±3.3	0.138
PaO_2_/ FiO_2_ ratio	195.3±35.1	210.1±76.5	0.412
Creatinine (umol/L)	101.6±48.6	97.6±31.2	0.736
BUN (mmol/L)	10.6±12.8	8.2±3.9	0.411
Total billirubin (umol/L)	19.6 ±9.6	19.4 ±10.6	0.949
ALT (IU/L)	47.4±84.7	64.1±119.9	0.537
AST (IU/L)	48.2±84.7	40.0±34.0	0.678

**AADA**, acute aorta dissection type A; **ALT**, alanine aminotransferase; **ASA**, American Society of Anesthesiologists; **AST**, aspartate aminotransferase; **BUN**, blood urea nitrogen; **DHCA**, deep hypothermic circulatory arrest; **MHCA**, modified hypothermic circulatory arrest; **LVEF**, left ventricular ejection fraction.

### Operative techniques

After induction of anaesthesia, the left radial artery ± one of the femoral arteries were cannulated for continuous measurement of systemic perfusion pressures during cardiopulmonary bypass. Temperature probes were placed for nasopharyngeal and rectal temperature monitoring. Intraoperative transesophageal echocardiography was used in all patients.

The patient was positioned on the operating table in a supine position. After administration of 300 IU/Kg heparin, an 8- or 10-mm Gore-Tex graft (Gore & Associates Inc., Arizona, USA) was anastomosed end-to-side to the right axillary artery with a running 6/0 polypropylene suture. The graft was then cannulated with a 22F elongated arterial cannula (Medtronic Corp, Minneapolis, MN) and secured.

All patients were approached through a midline sternotomy, extending the skin incisions a few centimeters above the manubrium. The anterior surface of the head vessels was carefully dissected. The decision for one particular cannulation strategy was made on the basis of preoperative investigations or intraoperative transesophageal echocardiography. When axillary artery alone was not suitable for arterial cannulation or failed to maintain adequate CPB flow, other arterial accesses in combination with or without axillary artery were considered, including the femoral artery, the brachiocephalic artery and the left common carotid artery.

The superior vena cava and inferior vena cava (IVC) were cannulated and drained separately for systemic venous return. CPB was started with a mean flow of 50-60 ml/kg/min. Systemic cooling was performed using the alpha-stat method for acid-base management. We continued to cool the patient and kept the mixed venous oxygen saturation at 75%-85%.

After reaching the target temperature, the patient was placed in Trendelenburg position, and the head was cooled topically. Selective unilateral ACP was initiated by clamping the brachiocephalic trunk before circulatory arrest and opening of the aorta. The extent of resection was determined by the extent of the aortic pathology and the location of entry site or reentry tear. If the aneurysm was confined to ascending aorta, only this segment was resected and a simple open distal anastomosis was performed. If the aortic arch had aneurysmal involvement, partial or total arch replacement was performed with or without an elephant trunk procedure. Upon completion of the circulatory arrest, the grafts were de-aired prior to restitution of full body perfusion and rewarming. Proximal aortic reconstruction as well as any concomitant procedures was conducted during the rewarming period.

### Temperature control and cardiopulmonary bypass management

In the DHCA group, patients received unilateral ACP with a constant flow rate of 8-10 ml/kg/min to maintain a perfusion pressure of 35-70 mmHg. During circulatory arrest, the nasopharyngeal temperature was kept at 18-20°C and rectal temperature was kept at 24°C-25°C. Patients in the MHCA group were managed with moderate hypothermia (nasopharyngeal temperature 24°C-25°C and rectal temperature 27°C-28°C), selective ACP (7-12 ml/kg/min with perfusion pressure maintained at 45-55 mmHg) and episodes of systemic retrograde perfusion during circulatory arrest (2-3 minutes per time with a flow of 5-8 ml/kg/min in every 15-20 minutes). The systemic retrograde perfusion was carried out through the IVC cannulation. After snaring the IVC by surgeons, perfusionist partially clamped the IVC return line to maintain the central venous pressure as high as 10-12 mmHg. As the arterial pressure of the lower body was less than 5 mmHg during the circulatory arrest, the elevated central venous pressure was adequate to drive a short period of the systemic retrograde perfusion. A 15%-20% drop of saturation of venous oxygen was regarded as an efficient retrograde perfusion.

### Definitions

Definitions were according to the Society of Thoracic Surgery National Database specifications (http://www.sts.org/sites/default/files/documents/word/STSAdultCVDataSpecificationsV2_73%20with%20correction.pdf). Emergent surgery was regarded as operation performed within 24 hours of hospital admission for cardiovascular instability. Acute aortic dissection is defined as occurring within 2 weeks of onset of initial symptoms [13]. Neurologic outcome was categorized as permanent neurologic dysfunction (PND) and temporary neurologic dysfunction (TND) [8,14]. PND was defined as the presence of a new postoperative focal (stroke) or global (Parkinsonism, coma, gait disturbance) neurologic deficit and persisting at discharge. TND was regarded as the occurrence of postoperative agitation, confusion, delirium, obtundation or a transient focal neurologic deficit (resolution within 72 hours) without any evidence of new structural abnormality on computed tomography or magnetic resonance imaging.

### Statistical analysis

Continuous variables in the tables and texts are expressed as mean ± standard deviation and categorical data as proportions. Categorical variables were compared using Chi-square test or Fisher’s exact test. Independent continuous variables were compared by unpaired Student’s *t* test for normally distributed data between two groups, and Mann-Whitney U-test was used for the comparison of parameters that did not exhibit a normal distribution. Variables with a univariate *p*<0.2 or those of known clinical importance for 30-day mortality and postoperative outcome events were included in a stepwise multivariate logistic regression model to calculate odds ratios (OR) and its 95% confidence intervals (CI) (Table 2). As others did before [15], we combined re-exploration for bleeding, dialysis-dependent renal failure, postoperative pneumonia, mediastinal infection, sepsis, tracheotomy and TND as the composite postoperative complications to increase the sensitivity for identifying risk factors. All *p* values less than 0.05 were considered statistically significant. All statistical analyses were performed using Statistical Package for Social Sciences (SPSS) version 16.0 (SPSS Inc, Chicago, IL, USA).

**Table 2:** Preoperative factors used in logistic regression analyses

**Preoperative factors**	**Intraoperative factors**
Age	Duration of cardiopulmonary bypass
Gender	Cross-clamp time
Hypertension	Circulatory arrest time ≥ 40 minutes
Diabetes	Core temperature during circulatory arrest
Left ventricular ejection fraction	Deep hypothermic circulatory arrest
ASA classification	Modified hypothermic circulatory arrest
Preoperative PaO_2_/FiO_2_ Ratio	
Renal insufficiency	
Liver function impairment	

**ASA**, American Society of Anesthesiologist.

## Results

A total of 54 patients underwent emergency surgery for AADA. The underlying chronic pathology was aneurysmal disease (88.9%), Marfan syndrome (9.3%) and other etiology (1.8%), such as infection or porcelain aorta. The mean age of patients in the series was 45.7±11.2 years (range, 22 to 75 years), 43 were male and 11 were female. Twenty-two (40.7%) patients and thirteen patients (24.1%) had a history of hypertension and diabetes, respectively. Hypothermic circulatory arrest and unilateral ACP were used in all patients and 21 patients in MHCA group underwent additional episodes of systemic retrograde perfusion during circulatory arrest.

### Cardiopulmonary bypass data and operative details

The mean CPB time in all patients was 256.0±84.62 minutes (range 131 to 566 minutes) and the mean aortic cross-clamp time was 149.5±41.6 minutes (range 62 to 294 minutes). The mean circulatory arrest time was significantly longer for patients in DHCA group than for patients undergoing MHCA (44.5 minutes versus 32.1 minutes, p=0.005; Table 3). Forty patients (74.1%) underwent total arch replacement, including 35 patients with a stage I elephant trunk procedure. Thirty patients (55.6%) underwent ascending aorta replacement, and one patient (3%) underwent hemiarch replacement. Proximal aortic procedures included Bentall procedure in 15 patients (27.8%), Cabrol procedure in 6 patients (11.1%), and Wheat procedure (1.9%). As demonstrated in Table 3, the different arch interventions equally distributed between the two groups. An additional 2 patients (3.7%) underwent aortic valve reconstruction and 2 patients (3.7%) received concomitant mitral valve interventions.

**Table 3:** Cardiopulmonary bypass data and surgical procedures

**Intraoperative data**	**All**	**DHCA**	**MHCA**	*p Value
	**(n=54)**	**(n=33)**	**(n=21)**	
Temperature during HCA				
Nasopharynx (°C)	20.3±3.9	17.6±2.4	24.6±0.4	<0.001
Rectal (°C)	22.5±3.8	19.7±2.3	26.4±1.1	<0.001
Operative time(minutes)	511.7±108.3	534.2±95.9	476.5±119.3	0.056
Duration of CPB (minutes)	256.0±84.3	289.5±79.9	203.3±61.9	<0.001
Cross-clamp (minutes)	149.5±41.6	161.9±34.9	130.2±44.6	0.005
Circulatory arrest (minutes)	39.6±16.6	44.5±18.4	32.1±9.7	0.006
Surgical Procedures				
Total aortic arch replacement	40(74.1%)	26(78.8%)	14(66.7%)	0.434
Elephant trunk	35(66.7%)	22(66.7%)	13(61.9%)	0.524
Ascending aorta replacement	30(55.6%)	17(51.5%)	13(61.9%)	0.433
Bentall procedure	15(27.8%)	8(24.2%)	7(33.3%)	0.399
Cabrol procedure	6(11.1%)	5(15.2%)	1(4.8%)	0.276

**CPB**, cardiopulmonary bypass; **DHCA**, deep hypothermic circulatory arrest; **MHCA**, modifies hypothermic circulatory arrest. *DHCA versus MHCA.

### Perioperative complications and outcome events

The overall incidence of TND was 9.2% and no patients had permanent neurologic deficits. The number of patients developing complicating pneumonia were significantly greater in the DHCA group than those in the MHCA group (57.6% versus 23.8%, *p=*0.015). Although the incidence of reexploration for bleeding was not significantly different between groups, the need for blood product transfusion in MHCA patients was significantly less as compared to the patients in the DHCA group (Table 4). Dialysis was required postoperatively in 6 patients (11.1%). Unadjusted differences between the two groups were not statistically different. Multivariate analysis identified age (OR, 2.74; 95% CI, 2.12 to 3.76; *p*=0.004) and CPB times (OR, 1.98; CI, 1.82 to 2.44; *p*=0.015) as independent predictors for composite complications, and the use of MHCA was determined as a protective factor against the composite postoperative complications (OR, 0.78; CI, 0.52 to 0.98; *p*=0.04).

**Table 4:** Postoperative morbidity and mortality

**Clinical characteristics & outcomes**	**DHCA**	**MHCA**	***p *Value**
	**(n=33)**	**(n=21)**	
In-hospital mortality	9(27.3%)	3(14.3%)	0.329
30-day mortality	11(33.3%)	4(19.0%)	0.253
Re-exploration for bleeding	2(6.1%)	4(19.0%)	0.193
Renal failure-dialysis	5(15.2%)	1(4.8%)	0.386
Pneumonia	19(57.6%)	5(23.8%)	**0.015**
Tracheotomy	13(39.4%)	2(9.5%)	**0.017**
Mediastinal infection	3(9.1%)	1(4.8%)	1.000
Sepsis	3(9.1%)	0(0%)	0.282
TND	4(12.1%)	1(4.8%)	0.638
PND	0 (0%)	0(0%)	1.000
Length of ICU stay, days	7.4±4.5	4.5±1.9	**0.010**
Length of hospital stay, days	18.6±6.2	17.4±5.5	0.556
Mechanical ventilation, hours	141.2±125.4	75.1±68.0	**0.035**
Red blood cell transfusion, IU	14 .6±8.2	10.3±7.3	**0.045**
FFP transfusion, ml	2590.9±1479.0	1419.1±1061.5	**0.003**
Platelet transfusion, IU	1.6±0.9	0.8±1.1	**0.007**
Drainage during first72h, ml	2040.2±1075.0	1543.8±1071.0	0.116
PaO_2_/ FiO_2_Ratio			
Postoperative day 1	170.0±43.9	163.5±40.7	0.718
Postoperative day 3	177.1±56.3	228.8±79.9	**0.017**
Lactate (mmol/L)			
Postoperative 1 hours	9.3±4.9	10.00±4.8	0.410
Postoperative 4 hours	5.9±3.8	5.9±3.4	0.983
Creatinine (umol/L) day 3	249.2±237.7	203.5±178.7	0.480
BUN (umol/L) day 3	25.5±14.6	23.0±13.1	0.538
Total bilirubin (umol/L) day 3	53.9±59.0	35.3±20.2	0.226
ALT (IU/L) day 3	103.2±91.4	102.0±108.0	0.967
AST (IU/L) day 3	97.6±107.7	78.6±70.1	0.502

**TND**, temporary neurologic dysfunction; **PND**, permanent neurologic dysfunction; **ICU**, intensive care unit; **BUN**, blood urea nitrogen; **FFP**, fresh frozen plasma; **ALT**, alanine aminotransferase; **AST**, aspartate aminotransferase.

The lactate level early after surgery in both groups was not significantly different (DHCA group: 9.27±4.93 mmol/L versus MHCA group: 9.98±4.75 mmol/L; *p*=0.41). Similarly, no differences in the parameters of renal and liver function, including creatinine, blood urea nitrogen, total bilirubin, and aminotransferases, in DHCA and MHCA patients were observed (Table 4). However, in patients who underwent circulatory arrest for more than 40 minutes, the renal and liver function of MHCA patients (n=9) was significantly improved as compared to the parameters in DHCA patients (n=15) (Table 5).

**Table 5:** Visceral function in patients with prolonged circulatory arrest

**Parameters**	**DHCA**	**MHCA**	***p *Value**
	**(n=15)**	**(n=9)**	
Lactate (mmol/L)			
Postoperative 1 hours	9.5±3.7	10.0±3.0	0.09
Postoperative 4 hours	6.1±2.9	5.8±3.4	0.32
Creatinine (umol/L) day 3	328.8±281.6	233.1±186.1	**0.007**
BUN (umol/L) day 3	27.1±10.6	23.9±9.1	**0.044**
Total bilirubin (umol/L) day 3	55.0±26.3	36.6±23.4	**0.010**
ALT (IU/L) day 3	119.2±34.7	96.5±49.8	**0.039**
AST (IU/L) day 3	97.8±82.6	81.3±69.0	**0.018**

**BUN**, blood urea nitrogen; **ALT**, alanine aminotransferase; **AST**, aspartate aminotransferase.

### Risk factors for prolonged hospitalization and early mortality

The average hospital stay was 18.1± 5.9 days, and no significant difference between the DHCA group and the MHCA group was observed (18.6±6.2 days versus 17.4±5.5 days, *p*=0.556). However, the average intensive care unit (ICU) stay was significantly shorter for patients in the MHCA group than patients from the DHCA group (4.5±1.9 days versus 7.4±4.5 days, *p*=0.010). Compared with patients who underwent deep hypothermia, patients in the MHCA group are less likely to have tracheotomy (9.5% versus 39.4%, *p*=0.017) and require shorter time of mechanical assisted ventilation (75.1±68.0 hours versus 141.2±125.4 hours, *p*=0.035). The use of MHCA was determined to be the only protective factor against the prolonged ICU stay by multivariate regression analysis (OR, 0.8; 95% CI, 0.56 to 0.98; *p*=0.04).

In-hospital mortality observed in our study was 22.2% (n=12), comparable to the published data from other similar case series [1,4,16,17]. Thirty-day mortality for the entire cohort was 27.8% (n=15) and no significant difference between groups was observed (p=0.263). Multivariate analysis identified preoperative American Society of Anesthesiologists classification (OR, 3.1; 95% CI, 1.062 to 9.258; *p*=0.019), duration of CPB (OR, 1.011; 95% CI, 1.002 to 1.019; *p*<0.05) as independent predictors of postoperative 30-day mortality.

## Discussion

For past two decades, DHCA in combination with either continuous antegrade or retrograde cerebral perfusion has gained its popularity due to the improved postoperative outcomes as compared with DHCA alone. As the real benefits of retrograde cerebral perfusion are not formally accepted, the combination of deep hypothermia and ACP during circulatory arrest was applied in our hospital (since 2007) and many other high-volume aortic centers. In recent years, the “warmer” temperature at circulatory arrest has been preferred as the safety of mild-to-moderate hypothermic circulatory arrest and the beneficial effects of avoiding deep hypothermia have been reported by a series of studies [6-10]. Since 2010, circulatory arrest in our institution has been obtained at a core temperature of 26°C-28°C. However, the avoidance of deep core temperature may be at the expense of an increased risk of the ischemic injuries of visceral organs and spinal cord. Thus, we modified our perfusion protocol by adding a short duration of retrograde systemic perfusion during circulatory arrest.

In the current study, we focused on assessing the effectiveness of our modified perfusion protocol (moderate hypothermia 26.4±1.1°C, ACP and retrograde systemic perfusion) for cerebral and visceral protection in the emergent repair of AADA, by comparing outcomes with a cohort that underwent similar surgical interventions at deep hypothermia (19.7±2.3°C). Analysis of the entire study population demonstrated that patients undergoing AADA surgery at moderate levels of hypothermia had a trend of reduced in-hospital and 30-day mortality compared with the patients in the DHCA group, but the difference was not statistically significant. The duration of CPB and preoperative American Society of Anesthesiologists classification were identified as independent predictors for early mortality in this study, similar to data published by Czerny and colleagues [18]. Several risk factors for postoperative mortality that have been identified by other studies, including age, preoperative respiratory failure (oxygenation index <200), renal insufficiency, level of hypothermia, left ventricular ejection fraction [2,4-12,19], were not found to be the significant predictors for 30-day mortality in our series.

The occurrence of neurological dysfunction after aortic surgery has been shown to be associated with longer ventilator requirements, prolonged ICU stay and higher in-hospital mortality [3,5]. According to the published literature, the overall incidence of TND in AADA patients undergoing hypothermic circulatory arrest and selective cerebral perfusion varies between 2.5-16% and PND with a reported incidence of 4-24% [2,12,15-17,19]. The risk of postoperative neurologic dysfunction was reported to be significantly influenced by the site of arterial cannulation and could be greatly reduced by cannulating a graft sewn to the right axillary artery [5,7,15]. All patients in this study were cannulated through an 8-10 mm Gore-Tex graft sewn to the axillary artery and then received ACP. Therefore, a low postoperative TND rate (9.5%) with no case of PND observed in our series was not unexpected. Previous studies have identified predictors of the negative neurologic outcomes in surgically treated AADA patients as age, history of cerebrovascular adverse events, renal insufficiency, prolonged hypothermic circulatory arrest time (>40 minutes), and CPB time [2,4,12,15,18,19]. However, in the current study, these risk factors were not found to be independent predictors for postoperative neurologic dysfunction. The reason for this might be attributed to the small sample size of our series, and probably, the surgical procedures in almost all cases were completed within the safety time limits during circulatory arrest.

The reduction of ventilator requirements in the MHCA patients translated into a significant shortened ICU length of stay compared with patients in the DHCA group. The reduction of ventilation requirements may be largely due to the significant shorter CPB times observed in the MHCA group (203.3±61.9 minutes) as compared to the DHCA group (289.5±79.9 minutes; *p*<0.001). It is well known that the extensive cooling and rewarming periods in deep hypothermia not only contributed to the longer duration of CPB and subsequent deep hypothermia-related vasoconstriction, coagulopathy, but also leads to a secondary vasodilation with increased interstitial edema and reperfusion injury during the extended rewarming phase [11]. Therefore, as we speculated, longer CPB time-induced pulmonary edema and prolonged mechanical ventilation may contribute to the higher incidence of postoperative pneumonia in patients underwent deep hypothermia (DHCA group: 57.6% versus MHCA group: 23.8%; *p*=0.015). Although the rate of reexploration for bleeding between the two groups was not significantly different, the requirements for blood product transfusion in DHCA patients were markedly higher compared with the MHCA group. This result suggests that the use of deep hypothermia was more likely to be associated with the disturbance of patients’ coagulation system.

**Figure 1:**
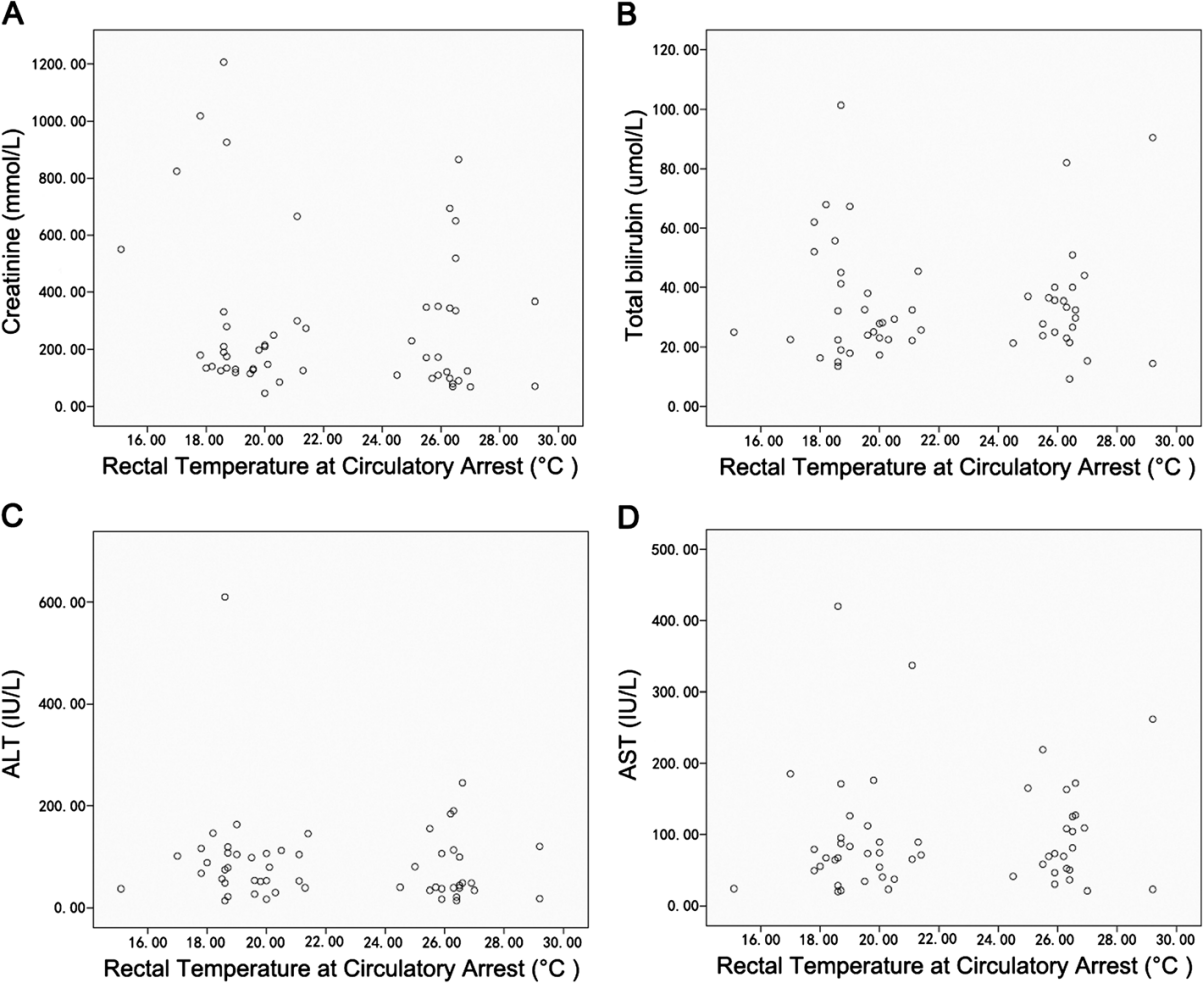
**The relationship between visceral organ function and core temperature at circulatory arrest.** Scatter plots showing no significant relationship between the early postoperative level of creatinine (**A**), total bilirubin (**B**), alanine aminotransferase (ALT, **C**), and aspartate aminotransferase (AST, **D**), and rectal temperature at circulatory arrest.

Theoretically, a cooler temperature could be related with better visceral organ protection, especially when a prolonged duration of lower body circulatory arrest is required. However, from our results, the postoperative renal and liver function in patients underwent moderate hypothermia were similar to those in the DHCA patients. As demonstrated in the scatter plots (Figure 1), no significant relationship between the rectal temperatures at circulatory arrest and the level of creatinine, total bilirubin and aminotransferases were observed. We suggest that in the DHCA group, the longer duration of CPB and the higher requirements for blood products transfusion may outweigh the beneficial effects provided by the lower temperature. Moreover, all patients in the MHCA group have received episodes of systemic retrograde perfusion during circulatory arrest. It is well known that human venous valves are largely distributed in veins in muscle-skeletal system rather than in visceral organs. During retrograde perfusion, the venous blood would firstly redistribute to veins without valves, such as venous system in liver, kidney and spinal cord, and therefore a better protection of these visceral organs in lower body might be achieved. Although the potential benefit of this method for lower body protection remains to be confirmed by large randomized trials, we may speculate its beneficial effects from the results of our series that the postoperative lactate level in MHCA patients was not inferior to that in DHCA patients, and visceral organ function in MHCA patients who underwent hypothermic circulatory arrest for longer than 40 minutes was significantly improved as compared with the parameters in the DHCA group.

The main limitations of the current study are its retrospective nature, and the absence of a control group of patients who underwent moderate hypothermic circulatory arrest without systemic retrograde perfusion. We acknowledge that without a direct comparison, the real benefits of our modified perfusion strategy for lower body protection remains to be confirmed. However, our results have indicated that AADA patients could benefit from “warmer” hypothermia during circulatory arrest. Furthermore, we cannot exclude the possibility that the improved surgical expertise might have an effect on outcomes, and a propensity score analysis with an enlarged sample size might be performed to limit any influence of the selection bias on our results.

## Conclusions

In summary, data from the current study support the safety and effectiveness of our modified perfusion strategy for cerebral and visceral organ protection during emergent repair of AADA. When directly compared with patients undergoing aortic reconstruction at deep hypothermia, no difference in early postoperative mortality, adverse neurologic outcomes and visceral organ dysfunction was observed. The reduction in blood product transfusion, respiratory complications and ICU stay suggest AADA patients might benefit more from the moderate hypothermia during circulatory arrest. Future studies will investigate the potential benefits of the systemic retrograde perfusion for lower body protection during circulatory arrest.

## Abbreviations

AADA: Acute aortic dissection type A; ACP: Antegrade cerebral perfusion; CPB: Cardiopulmonary bypass; DHCA: Deep hypothermic circulatory arrest; ICU: Intensive care unit; IVC: Inferior vena cava; MHCA: Modified hypothermic circulatory arrest; PND: Permanent neurologic dysfunction; TND: Temporary neurologic dysfunction.

## Competing interests

The authors declare that they have no competing interests.

## Authors’ contributions

All manuscript was written by HQ and JH, and all the data were collected by the following surgeons and perfusionists, HQ, JH, LD, YX, WM and EYZ. The collected data were promised by all authors. All authors read and approved the final manuscript.
